# Identification of a novel *KAT6A* variant in an infant presenting with facial dysmorphism and developmental delay: a case report and literature review

**DOI:** 10.1186/s12920-021-01148-x

**Published:** 2021-12-20

**Authors:** Soyoung Bae, Aram Yang, Jinsup Kim, Hyun Ju Lee, Hyun Kyung Park

**Affiliations:** 1 Department of Pediatrics, Hanyang University Medical Center, Hanyang University College of Medicine, 222-1, Wangshimri-ro, Sungdong-gu, Seoul, 04763 Republic of Korea; 2 Department of Pediatrics, Kangbuk Samsung Hospital, Sungkyunkwan University School of Medicine, Seoul, Republic of Korea

**Keywords:** Case report, Facial dysmorphism, Cleft palate, *KAT6A*, Arboleda-Tham syndrome

## Abstract

**Background:**

Arboleda-Tham syndrome (ARTHS), caused by a pathogenic variant of *KAT6A*, is an autosomal dominant inherited genetic disorder characterized by various degrees of developmental delay, dysmorphic facial appearance, cardiac anomalies, and gastrointestinal problems.

**Case presentation:**

A baby presented multiple facial deformities including a high arched and cleft palate, with philtral ridge and vermilion indentation, a prominent nasal bridge, a thin upper lip, low-set ears, an epicanthal fold, and cardiac malformations. Whole exome sequencing (WES) revealed a heterozygous nonsense mutation in exon 8 of the *KAT6A* gene (c.1312C>T, p.[Arg438*]) at 2 months of age. After a diagnosis of ARTHS, an expressive language delay was observed during serial assessments of developmental milestones.

**Conclusions:**

In this study, we describe a case with a novel *KAT6A* variant first identified in Korea. This case broadens the scope of clinical features of ARTHS and emphasizes that WES is necessary for early diagnosis in patients with dysmorphic facial appearances, developmental delay, and other congenital abnormalities.

**Supplementary Information:**

The online version contains supplementary material available at 10.1186/s12920-021-01148-x.

## Background

Arboleda-Tham syndrome (ARTHS, MIM 616268) is a newly defined, rare genetic disease caused by a pathogenic variant of *KAT6A* (MIM 601408) with autosomal dominant inheritance [1, 2]. Exome sequencing in patients with syndromic features associated with developmental delay and intellectual disability has revealed several causative pathogenic variants of *KAT6A* that are involved in the regulation of transcriptional activity and gene expression [1, 3–10]. The *KAT6A* gene, located on chromosome 8p11.21 and composed of 18 exons (KAT6A-201; ENST00000396930.3), encodes lysine (K) acetyltransferase 6A (KAT6A), which participates in chromatin remodeling, transcriptional regulation, and cellular replication [11, 12]. The major features of this syndrome include developmental delay, facial dysmorphism, microcephaly, cardiac anomalies, and gastrointestinal problems [5]. The frequent dysmorphic facial appearances in ARTHS include a prominent nasal bridge with a broad nasal tip, a thin-tented upper lip, and low-set ears [5, 7]. Most patients with pathogenic *KAT6A* variants have intellectual disabilities and speech delays that range from mild to severe. The genotype–phenotype analysis demonstrated that the truncating frameshift or nonsense variant within the penultimate and ultimate exons (16 and 17, respectively) is associated with severe developmental delay [5, 7, 13].

Here, we identified a novel likely pathogenic variant of *KAT6A* using whole exome sequencing (WES) analysis in an infant with a dysmorphic facial appearance, cleft palate, and minor cardiac malformations at the age of two months before a developmental delay was apparent. Early diagnosis with ARTHS such a young age, in which only facial dysmorphism was apparent before presenting with developmental delay, improved her cognitive outcome with intensive rehabilitation therapy.

## Case presentation

### Methods

#### Whole exome sequencing and Sanger sequencing

WES was performed in 3billion, Inc. (Seoul, South Korea) using a NovaSeq platform (Illumina, San Diego, CA, USA). Exome capture was performed with a Twist Human Core Exome Kit (Twist Bioscience, San Francisco, CA, USA). Raw genome sequencing data analyses included alignment to the reference sequence (National Center for Biotechnology Information genome assembly GRCh37; accessed in February of 2009). The mean read depth was 100-fold and 99.2% of the target region was covered. Variant calling, annotation, and prioritization were performed as previously described [14]. Clinical symptoms of cleft lip and palate (HP:0000202), facial dysmorphism (HP:0001999), and cardiac anomalies (HP:0001627) chosen for variant filtering of causative genes [15]. The identified variant was validated by Sander sequencing.

### Case report and result

**Fig. 1:**
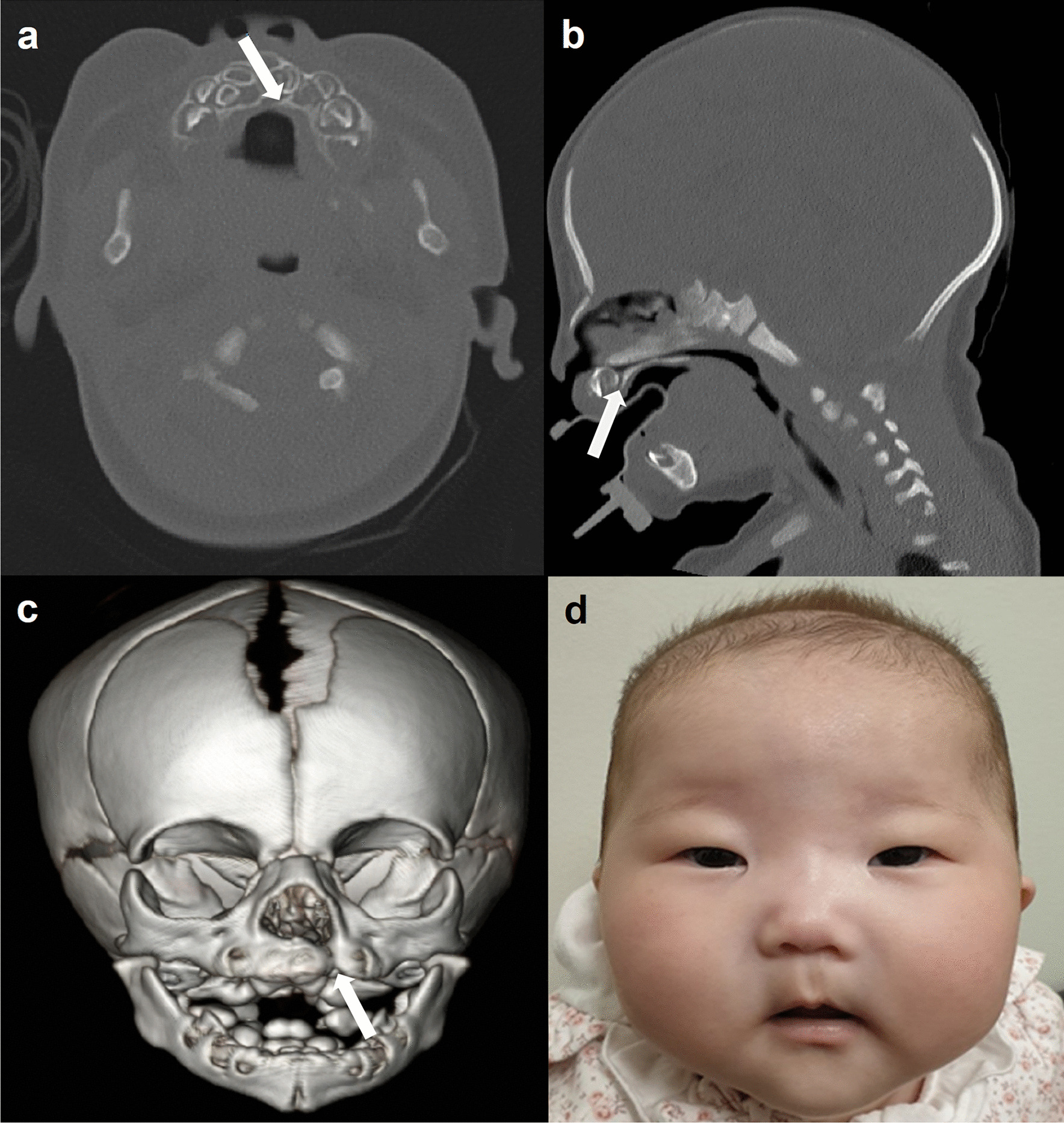
Facial computed tomography (CT) and photograph of the patient. **a** Axial, **b** coronal, and **c** three-dimensional reconstruction non-contrast CT images at three-weeks-of-age show unilateral bony defect (arrow) of the maxilla hard palate and mixed dentition. **d** Photograph of the patient

A newborn girl from healthy and non-consanguineous Korean parents was transferred to the neonatal intensive care unit at birth because of respiratory distress and desaturation. The infant was delivered at 40 + 2 weeks. The birth weight of the child was 3140 g (− 0.14 standard deviation [SD]), length was 52.3 cm (1.68 SD), and head circumference was 32.5 cm (− 1.17 SD). At birth, her mother and father were both 37 years old. Prenatal examinations, including non-invasive screening for aneuploidies, did not reveal abnormalities. During a one-month hospitalization for respiratory distress syndrome and persistent pulmonary hypertension, she presented with craniofacial deformities, including a high arched and cleft palate with philtral ridge and vermilion indentation, and a subtle dysmorphic facial appearance with a prominent nasal bridge, a thin-tented upper lip, low-set ears, and epicanthal fold (Fig. 1d). She showed no neurological abnormalities, such as hypotonia or spasticity, during hospitalization. Results of laboratory tests, including newborn screening tests for inborn errors of metabolism using tandem mass spectrometry, were normal. Although a thyroid hormone profile showed mild elevation of thyroid-stimulating hormone and free thyroxine, both recovered within one month. Minimal cleft lip with alveolar cleft was confirmed with facial computed tomography at age of three weeks (Fig. 1a–c). Depending on the size of the defect, the patient may require cheiloplasty at around age seven for cleft palate, which was classified as a unilateral incomplete form. Atrial septal defect (ASD) and small size (0.77 mm) patent ductus arteriosus (PDA) were observed via echocardiography three days after birth. The ASD closed on its own within one month. The device or surgical closure of PDA was not considered because of its small size requiring regular echocardiography. Results of the automated auditory brainstem response test, abdominal ultrasound, and ophthalmic examination were normal. Her brain magnetic resonance imaging, performed at three weeks of age, showed no structural abnormalities.

**Fig. 2:**
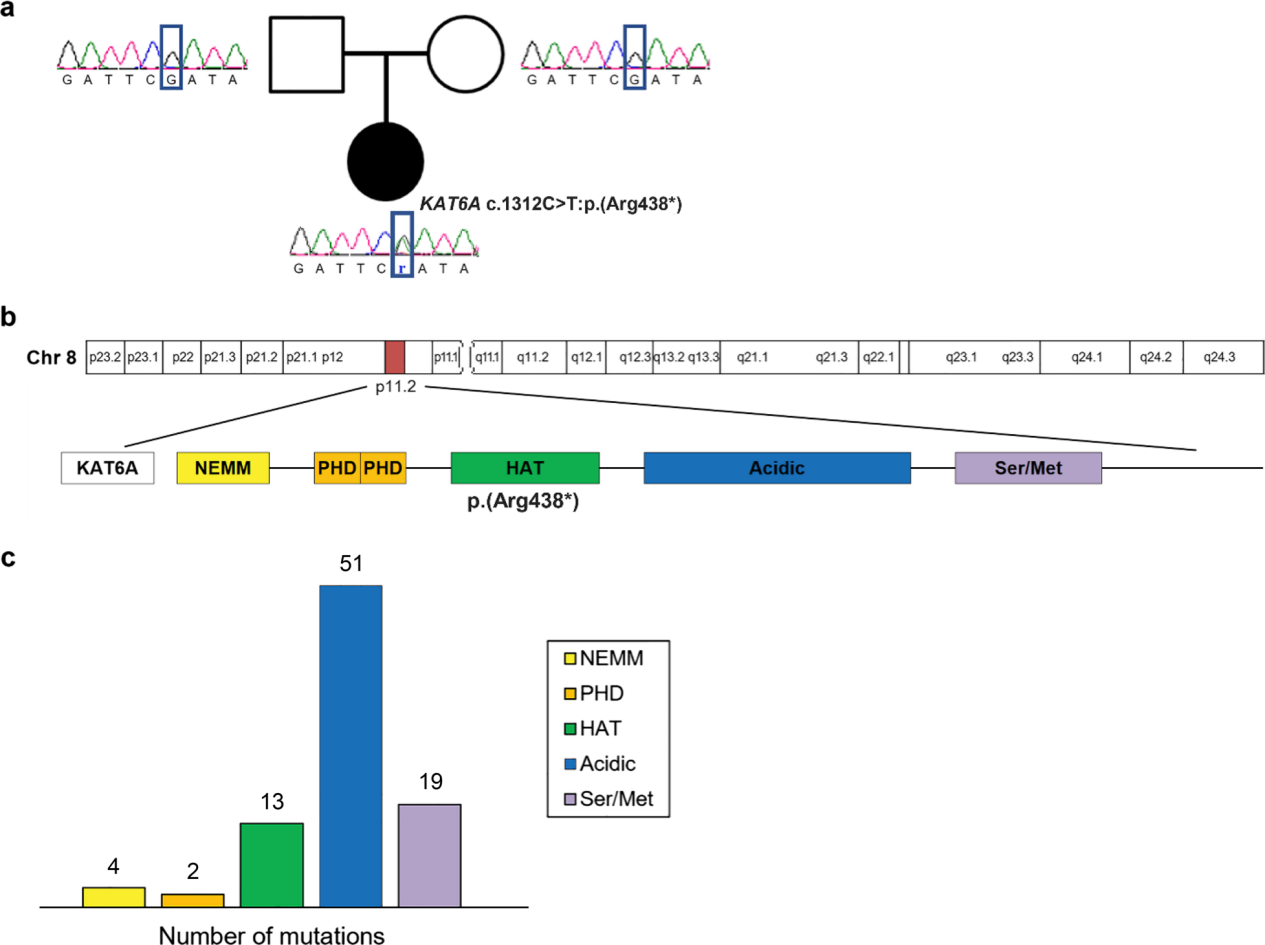
Localization of a pathogenic variant in *KAT6A*. **a** The family pedigree diagram with the electropherogram of Sanger sequencing. Heterozygote de novo mutation of c.1312C>T:p.(Arg438*) in exon 8 of the KAT6A gene was discovered by whole exome sequencing and was confirmed by Sanger sequencing analysis. **b** KAT6A protein domains and location of the novel variant in this study. **c** The number of previously reported mutations at each domain

She had a normal karyotype of 46, XX by high resolution (550-band level) karyotyping. WES was conducted when the patient was two months of age to search for pathogenic variants of genes causative of dysmorphic facial appearance, cleft palate, and cardiac anomalies **(**see Additional file 1**)**. The analysis showed a novel de novo heterozygous nonsense variant, Chr8:41834577G>A, *KAT6A*:NM_001305878.1:c.1312C>T:p.(Arg438*), in exon 8 of the *KAT6A* gene (Fig. 2a). The *KAT6A* variant was confirmed by Sanger sequencing. This nonsense was expected to cause protein truncation through nonsense-mediated mRNA, resulting in the loss of normal protein function. This variant was not detected in the several public genomic databases, such as The Genome Aggregation Database, the Exome Aggregation Consortium, 1000 Genomes, and the Exome Sequencing Project. Altogether, this variant was annotated as “likely pathogenic” according to the 2015 American College of Medical Genetics guidelines [16].

After diagnosis of ARTHS, developmental screening tests at the age of four months (Korean developmental screening test for infants and children; The Bayley Infant Neurodevelopmental Screener, 3^rd^ edition) did not indicate any developmental delays. However, a comprehensive developmental evaluation at the age of eight months with the Bayley Scales of Infant and Toddler Development, 3^rd^ edition (Bayley-III) found a mild language delay. Currently, the patient received a variety of intensive rehabilitation therapies, twice a week.

## Discussion and conclusions

Lysine acetyltransferase 6A, KAT6A, a member of the MYST family of proteins, forms a histone acetyltransferase complex and plays an essential role in regulating transcriptional activity and gene expression [17]. KAT6A is also involved in senescence regulation, neural stem cell proliferation, and cardiac septum development [12, 18]. Therefore, misregulation of KAT6A may result in tumorigenesis, intellectual disability, or congenital heart disease [18]. Since a recurrent translocation was noted in acute monocytic leukemia [19], pathogenic variants and misregulation of the human *KAT6A* gene have been identified in solid tumors and patients with syndromic disease with developmental disorders of variable severity [1, 2, 18, 20]. As WES has become a powerful means of investigating de novo pathogenic variants in neurodevelopmental disorders, the number of patients identified with novel genetic *KAT6A* variants has gradually increased [5, 7, 8, 13, 21, 22].

Since patients with syndromic developmental delay due to pathogenic variants of *KAT6A* were first reported in 2015, nearly 100 cases of ARTHS had delineated variable presentation, with a continuously expanding extent of symptoms [1, 2, 5, 7, 13]. We performed a literature review of 89 patients carrying de novo variants in *KAT6A*, including 34 frameshift mutations, 41 nonsense mutations, 9 missense mutations, 4 splicing mutations, and 1 deletion mutation (Table 1) [3–10]. Most of the reported mutations are loss of function and more than half of variants are located in the acidic domain (Fig. 2c) [5]. While the patients with late-truncating variants (defects in exon 16 or 17) displayed moderate-to-severe intellectual disabilities, early- truncations (exons 1–15) of *KAT6A* were mild [4, 5, 7]. The variant of this case is located in the HAT domain (Fig. 2b), which corresponds to an early truncating pathogenic variant. The phenotypic manifestations of ARTHS, including microcephaly, hypotonia, gastrointestinal problems and congenital heart defects, might be subtle in patients with early-truncating variants [5, 7].

**Table 1:** The genotypes and phenotypes of Arboleba-Tham syndrome [3–10]

	Kennedy et al. [5] (total 76)	Urreizti et al. [7]. (total 5)	Trinh et al. [4] (total 2)	Marji et al. [8] (total 1)	Alkhateeb et al. [6] (total 1)	Efthymiou et al. [3] (total 1)	Lin et al. [9] (total 1)	Jiang et al. [10] (total 1)	Patient	Overall (total 89)
			Number of subjects (%)							
Genotype										
Frameshift										
Early truncating	10	0	0	0	0	0	0	0	0	10/89 (11.2%)
Late truncating	19	2	0	1	1	0	0	1	0	24/89 (27.0%)
Nonsense										
Early truncating	7	0	0	0	0	0	1	0	1	9/89 (10.1%)
Late truncating	29	2	0	0	0	1	0	0	0	32/89 (36.0%)
Missense	6	1	2	0	0	0	0	0	0	9/89 (10.1%)
Splicing, late truncating	4	0	0	0	0	0	0	0	0	4/89 (4.5%)
Deletion, late truncating	1	0	0	0	0	0	0	0	0	1/89 (1.1%)
Phenotype										
Neurological problems										
Microcephaly	22	5	2	1	0	1	1	1	1	34/83 (41.0%)
Developmental delay (speech)	71	5	2	1	1	1	1	1	1	84/84 (100%)
Neonatal hypotonia	57	2	1	ND	1	1	1	0	0	63/86 (73.4%)
Craniofacial problems										
Broad/prominent nasal tip	57	4	2	ND	0	1	1	0	1	66/79 (83.5%)
Low-set ear	17	4	0	ND	0	0	1	0	1	23/40 (57.5%)
Cleft palate	3	0	0	ND	0	0	0	0	1	4/40 (10.0%)
Congenital heart problem										
ASD	25	2	0	ND	0	0	0	0	1	28/86 (32.6%)
VSD	6	0	0	ND	0	0	0	0	0	6/86 (7.0%)
PDA	14	0	0	ND	0	0	0	0	1	15/86 (17.4%)
Other problems										
Feeding difficulty	56	5	1	ND	1	ND	ND	ND	0	63/80 (78.7%)
Skeletal anomaly^*^	22	4	2	1	ND	ND	ND	ND	0	29/31 (93.5%)

^*^Skeletal anomalies include clinodactyly, camptodactyly, brachydactaly, arthrogyposis, scoliosis, torticollis, kyphosis, craniosynostosis, and congenital hip dysplasia

Percentages were calculated excluding unknown numbers. ND, not described; ASD, atrial septal defect, VSD, ventricular septal defect; PDA, patent ductus arteriosus

The spectrum of phenotypes with *KAT6A* variants includes neurological, craniofacial, gastrointestinal, cardiac, and ocular features. Neurological manifestations, such as developmental delay, hypotonia, microcephaly, and seizure, are main characteristic features of patients with ARTHS [4, 7]. All reported cases showed various degrees of developmental delays with frequent involvement of speech and expressive language (Table 1). In addition to speech delay, microcephaly, facial dysmorphism with a cleft palate, and cardiac problems were evident in this case. Microcephaly has been reported in 41.0% of patients (Table 1); however, it was less common in early-truncating variants [5, 7]. Most patients with ARTHS have a prominent nasal bridge with a broad nasal tip, a thin tented upper lip, and low-set, posteriorly rotated ears (Table 1). Other craniofacial features may be present, including frontal bossing, bitemporal narrowing, epicanthic folds, short and flat philtrums, and mild micrognathia [5, 7, 13]. Among craniofacial deformities, craniosynostosis was reported in 10% of patients with ARTHS, and cleft palate was noted in only three patients (Table 1) [5, 8]. Feeding difficulties were observed in 78.7% of patients with ARTHS (Table 1). Reflux, food allergies, and intestinal malrotation can also occur [5, 7, 22]. However, in patients with the early-truncating variant, gastrointestinal symptoms are less frequent [7]. Nearly half of patients with *KAT6A* variants have congenital heart disease, with ASD noted most frequently, followed by PDA (Table 1). The absence of gastrointestinal symptoms and minor cardiac anomaly shown in this case might be associated with the location of the variant. Other previously reported clinical features are strabismus, visual defects, joint hypermobility, syndactyly, supernumerary nipples, and cryptorchidism [7, 8, 20]. As more symptoms and comorbidities of ARTHS are discovered each time a new case is reported, it will be important to verify the genetic background of each case and to rule out the existence of other mild causal variants.

The salient features of this case included dysmorphic facial appearance, cleft palate, and congenital heart disease, which are major features that strongly suggest multiple congenital abnormalities [23–26]. As WES successfully identifies de novo, rare, and truncating genomic variants of several genes in undiagnosed cases with neurodevelopmental disorders or multiple congenital anomalies [21, 24, 27, 28]. Based on the cases reported and the database of the Deciphering Developmental Disorders Study, as many as 1% of undiagnosed syndromic developmental delays involve pathogenic *KAT6A* variants [1, 13]. WES also unravels the molecular basis of syndromic developmental delay associated with congenital neutropenia, food allergies, or multiple pituitary hormone deficiencies with malformation of the pituitary gland in patients with *KAT6A* variants [29–31]. Because of its broad phenotype spectrum, it is difficult to diagnose ARTHS based on clinical symptoms alone.

Genetic diagnosis at a younger age is important in syndromic cases because special treatments or surveillance programs are available for major symptoms. Most patients diagnosed with ARTHS have speech delays and intellectual disabilities, and early assessment of developmental delays can improve prognoses through proper treatment according to patient age [3–6]. In the present case, contrary to previous reports of patients with ARTHS, the genetic origin of the infant’s syndromic features was identified before an expressive language delay of mild degree developed at the age of eight months. At the time of diagnosis, the infant was only two months old, which is the youngest age reported to date, and developmental delays were not discernible at that age. She currently receives rehabilitation treatments twice a week and regular check-ups with Bayley-III at four-month intervals, which are expected to improve her cognitive impairment. As over 50% of patients experienced ophthalmologic abnormalities, including strabismus and visual abnormalities, regular visual assessments are required [5, 7]. Skeletal surveys are also needed because skeletal anomalies, including scoliosis, torticollis, and kyphosis, were found in 29 patients (Table 1).

Even though the role of *KAT6A* has not been clearly identified, the number of patients found to have de novo pathogenic variants of *KAT6A* continues to increase. Therefore, clinicians must be familiar with the various phenotypic features of ARTHS for the diagnosis by exome sequencing. In our case, because a genetic diagnosis was confirmed at an early age, the unexpressed features that may develop later in life can be monitored. Moreover, further study of the variant is important since there had been no functional studies to confirm that this variant was truly deleterious. This report broadens the scope of clinical features of ARTHS that might be an unrevealed genetic cause of neurodevelopmental disorder and facial dysmorphism. WES is useful to identify any associated genetic disorders and provide personalized care for patients with facial dysmorphism and other congenital anomalies possibly associated with developmental delay.

## Supplementary Information

**Additional file 1.** Candidate variants identified by whole exome sequencing

## Data Availability

The details of the variant analyzed during the current study are available in the ClinVar database repository (https://www.ncbi.nlm.nih.gov/clinvar/variation/1325410/). The raw datasets of participant generated during the current study are available in the Sequence Read Archive (SRA) database repository (https://www.ncbi.nlm.nih.gov/sra/?term=PRJNA788062/). Public databases used in this study included 1000genomes (http://www.1000genomes.org/), the Genome Aggregation Database (http://gnomad.broadinstitute.org), the Exome Aggregation Consortium (http://exac.broadinstitute.org/), the Exome Sequencing Project (http://evs.gs.washington.edu/), the Ensemble Project (http://ensemble.org/), the Human Phenotype Ontology (http://hpo.jax.org/), Human reference genome (GRCH37/hg19) (https://www.ncbi.nlm.nih.gov/assembly/GCF_000001405.13/), the DECIPHER database (https://www.deciphergenomics.org/), and OMIM (https://www.omim.org/).

## Abbreviations

ARTHS: Arboleda-Tham syndrome
ASD: Atrial septal defect
Bayley-III: Bayley Scales of Infant and Toddler Development, 3rd edition
CT: Computed tomography
IRB: Institutional Review Board
KAT6A: Lysine acetyltransferase 6A
ND: Not described
PCR: Polymerase chain reaction
PDA: Patent ductus arteriosus
VSD: Ventricular septal defect
WES: Whole exome sequencing
